# Detecting quantitative trait loci and exploring chromosomal pairing in autopolyploids using polyqtlR

**DOI:** 10.1093/bioinformatics/btab574

**Published:** 2021-08-06

**Authors:** Peter M Bourke, Roeland E Voorrips, Christine A Hackett, Geert van Geest, Johan H Willemsen, Paul Arens, Marinus J M Smulders, Richard G F Visser, Chris Maliepaard

**Affiliations:** Plant Breeding, Wageningen University & Research, 6708PB Wageningen, The Netherlands; Plant Breeding, Wageningen University & Research, 6708PB Wageningen, The Netherlands; Biomathematics and Statistics Scotland, Invergowrie, Dundee DD2 5DA, UK; Plant Breeding, Wageningen University & Research, 6708PB Wageningen, The Netherlands; Deliflor Chrysanten B.V., 2676BS Maasdijk, The Netherlands; Plant Breeding, Wageningen University & Research, 6708PB Wageningen, The Netherlands; Plant Breeding, Wageningen University & Research, 6708PB Wageningen, The Netherlands; Plant Breeding, Wageningen University & Research, 6708PB Wageningen, The Netherlands; Plant Breeding, Wageningen University & Research, 6708PB Wageningen, The Netherlands; Plant Breeding, Wageningen University & Research, 6708PB Wageningen, The Netherlands

## Abstract

**Motivation:**

The investigation of quantitative trait loci (QTL) is an essential component in our understanding of how organisms vary phenotypically. However, many important crop species are polyploid (carrying more than two copies of each chromosome), requiring specialized tools for such analyses. Moreover, deciphering meiotic processes at higher ploidy levels is not straightforward, but is necessary to understand the reproductive dynamics of these species, or uncover potential barriers to their genetic improvement.

**Results:**

Here, we present polyqtlR, a novel software tool to facilitate such analyses in (auto)polyploid crops. It performs QTL interval mapping in F_1_ populations of outcrossing polyploids of any ploidy level using identity-by-descent probabilities. The allelic composition of discovered QTL can be explored, enabling favourable alleles to be identified and tracked in the population. Visualization tools within the package facilitate this process, and options to include genetic co-factors and experimental factors are included. Detailed information on polyploid meiosis including prediction of multivalent pairing structures, detection of preferential chromosomal pairing and location of double reduction events can be performed.

**Availabilityand implementation:**

polyqtlR is freely available from the Comprehensive R Archive Network (CRAN) at http://cran.r-project.org/package=polyqtlR.

**Supplementary information:**

Supplementary data are available at *Bioinformatics* online.

## 1 Introduction

Polyploids, which carry more than two copies of each chromosome, are an important group of organisms that occur widely among plant species, including several domesticated crops (Salman-Minkov *et al.*, 2016). Many theories to explain their prevalence among crop species have been proposed, identifying features which may have appealed to early farmers in their domestication of wild species. Such features include their larger organs, such as tubers, fruits or flowers (the so-called ‘gigas’ effect) (Sattler *et al.*, 2016), phenotypic novelty (Udall and Wendel, 2006), their ability to be clonally propagated (Herben *et al.*, 2017), increased seedling and juvenile vigour (Levin, 1983) and the possibility of seedlessness which accompanies odd-numbered ploidies (Bradshaw, 2016). From a functional perspective, these features may be associated with factors such as increased heterosis (Comai, 2005), a greater level of genomic plasticity (te Beest *et al.*, 2012) or a masking effect of deleterious alleles (Renny-Byfield and Wendel, 2014). It is currently believed that all flowering plants have experienced at least one whole genome duplication (WGD) during the course of their evolution, with many lineages undergoing multiple rounds of WGD followed by re-diploidization (Vanneste *et al.*, 2014). Polyploidy may also be induced deliberately [through species hybridization with associated unreduced gametes, or through the use of some chemical cell division inhibitor, such as colchicine (Blakeslee and Avery, 1937)], often to combine properties of parents that could not otherwise be crossed (Van Tuyl and Lim, 2003), or to benefit from some of the other advantages listed above.

Polyploids are generally divided into two groups: autopolyploids, with multiple copies of the same homologous chromosomes derived from a single progenitor species, and allopolyploids, with multiple copies of homoeologous chromosomes from multiple progenitor species that continue to pair and recombine within but not between homoeologues. Allopolyploids are said to exhibit ‘disomic’ inheritance, i.e. genetically speaking they are equivalent to diploids. Autopolyploids are, on the other hand, genetically distinct from allopolyploids in that they exhibit ‘polysomic’ inheritance, a property that emerges from random pairing and recombination between homologues during meiosis. As most software and methodology for genetic analyses has traditionally been developed for diploid organisms, progress in autopolyploid breeding and research has been slower. In recent years this has gradually changed, as more tools become available for autopolyploids too (Bourke *et al.*, 2018c).

One of the greatest difficulties in autopolyploid cultivation and breeding is the constant re-shuffling of alleles in each generation, a consequence of polysomic inheritance. Breeders would like to be able to identify genomic regions that contribute favourable alleles to a particular trait of interest [quantitative trait loci (QTL)] and predict which offspring in a population carry favourable combinations of parental alleles. Understanding how genes and their alleles are transmitted from one generation to the next, or identifying potential barriers to recombination that might restrict allelic combinations from arising can provide insights into designing crosses and identifying favourable offspring from these crosses. The use of genomic information can greatly assist in these efforts. Particularly for polyploid species, specialized software tools are required for this purpose.

Polyploid genotyping involves the estimation of dosage [counts of the alternative allele at a polymorphic site, usually bi-allelic single nucleotide polymorphisms (SNPs)]. In an autotetraploid for example, the possible dosages range from nulliplex (0 copies of the alternative allele), simplex (1 copy), duplex (2 copies), triplex (3 copies) to quadruplex (4 copies). The assignment of marker dosage in polyploids is a non-trivial problem in itself, but there is an increasing number of possibilities for achieving this using dedicated software (Carley *et al.*, 2017; Clark *et al.*, 2019; Gerard *et al.*, 2018; Pereira *et al.*, 2018; Serang *et al.*, 2012; Voorrips *et al.*, 2011; Zych *et al.*, 2019).

Identity-by-descent (IBD) probabilities are the inheritance probabilities of parental alleles in a population of related genotypes (either bi-parental or multi-parental), and they can be exploited both for QTL mapping and to accurately interpret parental meiosis and inheritance patterns. Hidden Markov Models (HMM) have previously been applied to estimate these inheritance probabilities for polyploids (Hackett *et al.*, 2013; Mollinari and Garcia, 2019; Zheng *et al.*, 2016, 2020), and have been shown to be robust against common issues such as genotyping errors or local ordering issues in the underlying linkage maps (Zheng *et al.*, 2016). Of the currently available methods, both TetraOrigin and polyOrigin include a fully generalized polysomic model with the possibility of including multivalents in the model of parental meiosis (Zheng *et al.*, 2016, 2020). These packages are, however, currently aimed at tetraploid species. MAPpoly implements a HMM to estimate IBD probabilities that can be applied for all even ploidy levels, but assumes bivalent pairing only (Mollinari and Garcia, 2019). However, autopolyploids carry homologous chromosomes that often pair during meiosis in more complex structures called multivalents, associations of more than two homologues (generally only even numbers are considered viable). In particular, the phenomenon of double reduction, a possible product of multivalent pairing where both copies of a segment of sister chromatids are passed on to an offspring (Bourke *et al.*, 2015), is ignored. The overall impact of omitting double reduction from the model used for QTL analysis has previously been shown to be relatively minor in a QTL analysis that does not account for it (Bourke *et al.*, 2019), but double reduction events at specific loci may have important breeding implications (e.g. an offspring carrying a double copy of a favourable allele at that locus). For higher ploidy levels (6× and higher), HMM approaches may lead to computational bottlenecks (Mollinari and Garcia, 2019). Alternative approaches to estimate IBD probabilities have been proposed (Bourke, 2014) and although less accurate, have the advantage of being computationally tractable at higher ploidy levels and have previously been successfully used in the analysis of several traits in hexaploid chrysanthemum for example (Van Geest *et al.*, 2017a).

Apart from their application in QTL mapping, IBD probabilities provide a powerful approach to reconstruct meiotic processes and identify recombination events in polyploid individuals. This latter point can be exploited to address the issue of genotyping errors. They also yield insights into potential preferential chromosomal pairing, which is increasingly being acknowledged as a feature of many polyploid species that were previously assumed to be either purely auto- or allopolyploid (Bourke *et al.*, 2017; Leal‐Bertioli *et al.*, 2018). In this article, we describe the features of polyqtlR, a novel R package (R Core Team, 2020) for QTL mapping in both auto- and allopolyploid species which addresses many of the complexities of polyploid inheritance mentioned above. Estimation of IBD probabilities under a full polysomic model (including multivalents and double reduction) is performed for autotriploid, autotetraploid and autohexaploid F_1_ populations, while IBD probabilities of diploids and allopolyploids are estimated using a diploid HMM. Alternatively, a computationally efficient but approximate method for IBD estimation suitable for all ploidy levels (allo- and auto-) is implemented in polyqtlR. With these IBD probabilities, a range of applications are available, for QTL discovery and exploration as well as investigation of meiotic processes and patterns of recombination across the genome.

## 2 Materials and methods

### 2.1 Input data

polyqtlR requires as input dosage-scored marker information with an accompanying phased linkage map from an F_1_ population. Dosage scores can either be discrete or probabilistic (i.e. the probabilities of each of the dosage classes from 0 to *ploidy* for each individual at a marker), while phased linkage maps can be generated using software such as TetraploidSNPMap (Hackett *et al.*, 2017), polymapR (Bourke *et al.*, 2018b) or MAPpoly (Mollinari and Garcia, 2019). For hexaploid populations, only polymapR or MAPpoly are currently suitable, while polymapR is the only software that can also map odd-numbered ploidies such as triploid populations (Bourke *et al.*, 2018b). In the case of tetraploids for which a marker order is already known, parental map phase and IBD probabilities can also be estimated using TetraOrigin or polyOrigin (Zheng *et al.*, 2016, 2020).

### 2.2 Modelling autopolyploid meiosis

#### 2.2.1 Hidden Markov Model

The methodology behind the estimation of offspring IBD probabilities was originally developed for tetraploid populations (Zheng *et al.*, 2016) but we have extended the approach to a range of commonly encountered ploidy levels (2×, 3×, 4× and 6×). Details are contained in Supplementary Methods S1.

#### 2.2.2 Heuristic model

An algorithm for approximating IBD probabilities without using HMM is also implemented in polyqtlR. This uses an approach originally described inBourke (2014) and re-implemented by Van Geest et al. (2017a). Details are contained in Supplementary Methods S2. Finally, IBD probabilities may be interpolated at a regular grid of positions using cubic splines (by default at 1 cM spacings).

### 2.3 Form of the QTL model

The IBD-based QTL analysis uses a linear regression on the parental homologue probabilities, broadly similar to the weighted regression model proposed by Kempthorne (1957) and implemented in the TetraploidSNPMap software (Hackett *et al.*, 2013, 2014, 2017). For a tetraploid, the form of the model is:

$$Y=\;\mu +\alpha_{1}X_{1}+\alpha_{2}X_{2}+\alpha_{3}X_{3}+\alpha_{4}X_{4}+{\alpha_{5}X_{5}+\alpha }_{6}X_{6}+\alpha_{7}X_{7}+\alpha_{8}X_{8}+\epsilon$$

Here, the *X_i_* are inheritance probabilities for each parental homologue (1–4 for parent 1, 5–8 for parent 2 in a tetraploid), which range from $0\leq X_{i}\leq 1\;$when bivalent-only pairing is assumed. In the case where multivalents are also permitted in the meiotic model, more than one copy of a parental homologue can be inherited through the process of double reduction, in which case *X_i_* are the total sum of inheritance probabilities for each parental homologue, with $0\leq X_{i}\leq 2$.

In the context of a tetraploid, it can be generally assumed $X_{1}+X_{2}+X_{3}+X_{4}=2$ and $X_{5}+X_{6}+X_{7}+X_{8}=2$ (i.e. both parents contribute an equal number of chromosomes to an offspring). Eliminating $X_{1}$ and $X_{5}$ by substituting these expressions into the previous equation (to remove collinearity) leads to the following model:

$$Y=\;\mu +2\left(\alpha_{1}+\alpha_{5}\right)+\left(\alpha_{2}-\alpha_{1}\right)X_{2}+\left(\alpha_{3}-\alpha_{1}\right)X_{3}+\left(\alpha_{4}-\alpha_{1}\right)X_{4}+(\alpha_{6}-\alpha_{5})X_{6}+(\alpha_{7}-\alpha_{5})X_{7}+(\alpha_{8}-\alpha_{5})X_{8}+\epsilon$$

This can be re-written as:

$$Y=\;\mu^{'}+\alpha_{2}^{'}X_{2}+\alpha_{3}^{'}X_{3}+\alpha_{4}^{'}X_{4}+\alpha_{6}^{'}X_{6}+\alpha_{7}^{'}X_{7}+\alpha_{8}^{'}X_{8}+\epsilon$$

where $\mu^{'}$ is the adjusted intercept ($\mu^{'}=\;\mu +2\left(\alpha_{1}+\alpha_{5}\right)$), $\alpha_{i}^{'}$ are the adjusted regression co-efficients (e.g. $\alpha_{2}^{'}$ = $\alpha_{2}-\alpha_{1}$) and ϵ is the residual term. For a hexaploid, the model includes ten of the twelve parental homologues (Van Geest *et al.*, 2017a), etc.

A single marker analysis option is also included in the package, in which a genome-wide scan is performed by fitting the following additive model at each marker position:

$$Y=\bar{y}+\alpha D+\epsilon$$

where Y is the vector of phenotypes, D is the vector of marker scores, $\bar{y}$ is the overall mean and ϵ the residuals.

If experimental factors (loosely termed ‘blocks’ here, although they could correspond to different years, environments, etc.) are included, they are first fitted (Y ∼Blocks) after which the residuals are used to perform the genome-wide QTL scan. Missing phenotypes are imputed using fitted block effects and non-missing phenotype scores for that individual in other blocks. By default, at least 50% observations are required for imputation (e.g. minimum 2 out of 3 phenotypes non-missing for that individual in a 3-block situation). Estimating BLUEs [using a linear mixed model with genotypes as fixed effects (Pinheiro *et al.*, 2017)] for block-corrected trait values can speed up the analyses, particularly when estimating significance thresholds.

The sum of squared residuals (RSS_1_) is recorded from the ANOVA table (for both IBD-based and single marker approaches) and used to calculate the logarithm of odds ratio (LOD) score as follows (Broman and Sen, 2009):

$$\mathrm{LOD}=\;\frac{N}{2}\log_{10}\left(\frac{{\mathrm{RSS}}_{0}}{{\mathrm{RSS}}_{1}}\right)$$

where *N* is the population size, and RSS_0_ is the residual sum of squares under the Null (no QTL) Model. In cases where large-effect QTL are present and segregating in a population, it can be advantageous to reduce the level of background noise at other loci by accounting first for the major QTL and running an analysis on the QTL-corrected phenotypes. Such an approach has previously been termed multiple QTL mapping (Jansen, 1992, 1993). In polyqtlR, we follow a similar approach to correct for genetic co-factors, either by supplying the name of a marker closely linked to the major QTL peak, or the QTL peak position from the genome-wide scan (usually performed at regular intervals for efficiency). There is no limit to the number of co-factors that can be added, but a parsimonious analysis with only significant QTL as genetic co-factors is recommended (to avoid issues of collinearity). Automatic fitting of genetic co-factors is also implemented, fitting all possible combinations of initially detected QTL exceeding the significance threshold as co-factors (i.e. for QTL *q*_1_, *q*_2_, …, *q*_n_, all co-factor models Ui=1nC(qiin) are tested, where C(qi)in denotes all *i*-wise combinations of QTL for i∈[1,2,…,n]). Following this, a set of positions that individually maximized the threshold-adjusted LOD scores within the genetic region associated with each QTL locus are identified (a QTL locus is by default assumed to be no smaller than a 20 cM interval—i.e. this is the smallest assumed resolution between independent QTL that could occur). These new set of positions are then fed back into the same procedure to refine the estimates of QTL position and threshold-corrected significance, with positions that maximize the threshold-corrected LOD score being selected. Internally, the QTL model described above for IBD probabilities is initially fitted at the supplied position(s) and the residuals are saved to replace the vector of phenotype values in the QTL scan. Note that when blocks or genetic co-factors are included, they form part of the Null Model in the calculation of LOD scores.

The percentage of phenotypic variance explained at a single position is estimated by

$$\mathrm{PVE}=\;100*(1-\;10^{\frac{-2*\mathrm{LOD}}{N}})$$

In the case of a multi-QTL model, the PVE is estimated using

$$100*(1-\;\frac{{\mathrm{RSS}}_{1}}{{\mathrm{RSS}}_{0}})$$

where RSS_1_ is now recorded from the fitted (multi-) QTL model and RSS_0_ from the no-QTL model (Broman and Sen, 2009).

Approximate significance thresholds are determined using Permutation Tests (Churchill and Doerge, 1994). The number of permutations *N_p_* and the approximate Type I error rate *α* can be specified. By default *N_p_* = 1000 permutations of trait values are performed, after which the maximum genome-wide LOD scores are recorded from each of the *N_p_* genome-wide scans. The 100*(1 - *α*) percentile of the ordered LOD scores is taken as an approximate 100*(1 - *α*) % significance threshold (by default *α* = 0.05). Chromosome-specific thresholds can be generated by restricting the input to the chromosome(s) of interest, if so desired.

### 2.4 Exploration of QTL configuration, mode of action

One of the advantages of an IBD-based analysis over single-marker methods is the ability to explore QTL peak positions to determine the most likely QTL configuration (the parental origin of QTL alleles that have an effect on the phenotype), their mode of action (additive/dominant) and the effect sizes (both positive and negative) of specific parental alleles. A range of QTL models can be compared in polyqtlR using the Bayesian Information Criterion (BIC) (Schwarz, 1978) as previously proposed (Hackett *et al.*, 2014). Homologue-specific effects can be visualized around QTL peaks, aiding in the interpretation of the most likely predicted QTL configuration.

### 2.5 Genotypic information coefficient

The genotypic information coefficient (GIC) is a convenient measure of the precision of our knowledge on the composition of parental alleles carried by each offspring individual at a particular position, averaged across the mapping population. This is visualized in a similar manner to QTL profile plots, providing an overview of the genome-wide information landscape in the population in the vicinity of detected (or indeed expected but undetected) QTL.

The GIC of homologue *j* (1≤j≤ploidy1+ploidy2) at each position is calculated from the IBD probabilities using the formula:

$${\mathrm{GIC}}_{j}=\;1-\frac{4}{N}\sum_{n=1}^{N}P_{n,j}(1-P_{n,j})$$

where ploidy1 and ploidy2 refer to the ploidy levels of the two parents, *N* is the population size and $P_{n,j}$ is the probability that individual *n* inherited homologue *j* at that position [this is a generalization of the GIC measure used in MapQTL (Van Ooijen 1992, 2009)]; for a derivation see Appendix I of Bourke *et al.* (2019). When multivalents are included in the HMM, only offspring predicted to have come from bivalent-only meioses for that linkage group are used in the calculation.

### 2.6 Polyploid meiosis

Several aspects of polyploid meiosis can be investigated using IBD probabilities. These include detecting signatures of multivalent pairing structures in tetraploid and hexaploid parents, determining rates of double reduction, identifying recombinations from cross-overs, and looking for deviations from random pairing in meiosis, a feature associated with autopolyploidy (polysomic inheritance). Non-random chromosomal pairing, also called ‘preferential pairing’ (Bourke *et al.*, 2017) can be detected across a population using counts of bivalent pairing structures. Using the HMM method of IBD estimation, each valency (homologue pairing configuration) has an associated posterior probability. Deviations from random pairing are tested per parental chromosome using a chi-square test on the counts of predicted pairings.

### 2.7 Cross-overs, errors and linkage map curation

Recombinations from cross-overs are detected given a predicted pairing by looking for regions in which the inheritance probabilities of pairing parental homologues switch from one homologue to the other. A threshold probability is defined (by default 0.4) to identify meiotic pairing patterns that were clearly predicted or ‘plausible’ using the HMM and screen out those that were ambiguous. Within the context of bivalent pairing, each individual has an associated inheritance probability (IBD) for homologues in each bivalent pair. A recombination break-point is defined as a point at which the difference in inheritance probabilities of such pairing homologues changes sign. Its position is taken as the midpoint between the flanking positions for which such a switch-over in inheritance probabilities occurred. Individuals showing unexpectedly high numbers of recombinations can be identified and removed. One of the input parameters in the HMM method of IBD estimation is the error prior ε, the genome-wide error rate in the offspring genotypes. With high-quality data, error priors of the order 0.01–0.05 are reasonable, while for poorer-quality data a higher error prior may be required. If IBD probabilities are estimated using a suitably high error prior (ε=0.2, say), spurious recombinations from genotyping errors are suppressed, in which case IBD parental homologue probabilities can be used to directly re-impute marker genotype dosages with the function impute_dosages. For each individual, the imputed dosage of individual *j* at marker *n* on a certain linkage group is given by:

$${\hat{d}}_{n,j}=\sum H*P$$

where H is the (*ploidy1 *+* ploidy2*)×1 vector of parental homologue probabilities of that individual at that marker position (*ploidy1* and *ploidy2* being the ploidy levels of parent 1 and parent 2, respectively), P is the (*ploidy1 *+* ploidy2*)×1 vector of parental phase coded in 0 and 1 notation (1 for presence, 0 for absence) and H*P is their element-wise product. This operation generally leads to non-integer dosage values, and so ${\hat{d}}_{n,j}$ is rounded to the nearest integer. If the absolute value of the difference between the exact and rounded values exceeds a user-defined rounding error threshold (by default 0.05), the imputed dosage is set to missing.

## 3 Results

We demonstrate the capabilities of polyqtlR with a number of example applications. We first analysed an example trait in both tetraploid and hexaploid material and compared our results to those generated using TetraploidSNPMap (Hackett *et al.*, 2017) and QTLpoly (Pereira *et al.*, 2020). We then used the package to dissect the meiotic patterns of a hexaploid chrysanthemum population (Van Geest *et al.*, 2017a). Finally, we performed some tests to quantify the accuracy and computational performance of the IBD estimation module in the package.

### 3.1 QTL detection

**Fig. 1.**
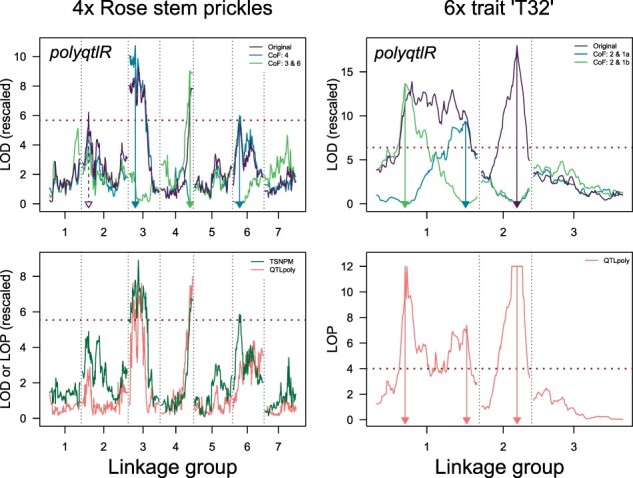
Comparison of the results of polyqtlR with those of alternative methods in both a tetraploid and hexaploid dataset. Upper panels: results of polyqtlR; Lower panels: results of TSNPM = TetraploidSNPMap and QTLpoly for two example traits: stem prickles in tetraploid rose (left panel) and trait ‘T32’ in a simulated hexaploid dataset (right panel). Estimated QTL positions are highlighted with arrows. In the case of the LG 2 QTL for stem prickles, no significant association was detected after fitting co-factors (dotted purple arrow). Legend ‘CoF: 3 & 6’ refers to a co-factor model with QTL positions on LG 3 and 6 included as co-factors. On the *y*-axes, LOD or LOP (-log_10_(*P*)) scores were re-scaled so that independently estimated significance thresholds overlap on the plot. For the trait ‘T32’, QTLpoly returned *P*-values of 0 around the QTL on LG 2 which cannot be visualized using LOP and were therefore artificially replaced, leading to a plateau around the peak

A tetraploid cut rose (*Rosa*×*hybrida*) dataset for the morphological trait ‘stem prickles’ using a previously published linkage map (Bourke *et al.*, 2017) and phenotypic data collected in different growing environments (Bourke *et al.*, 2018a) was analysed with polyqtlR, QTLpoly and TetraploidSNPMap. The first genome-wide scan for QTL using polyqtlR detected four putative QTL on LG 2, 3, 4 and 6 (Fig. 1). By fitting various combinations of QTL as co-factors, we found that the significance of the LG 3, 4 and 6 peaks could be increased, while the peak on LG 2 dropped in significance upon the inclusion of co-factors. Different co-factor combinations were tested for each QTL. The analysis that resulted in the highest threshold-adjusted LOD score for that peak was used to estimate the QTL position. The four-QTL model was found to explain 54% of the phenotypic variation (PVE), while the best three- and two-QTL models explained 49% and 43% of the phenotypic variation, respectively. TetraploidSNPMap predicted a three-QTL model, detecting the same QTL on LG 3, 4 and 6, while a putative position on LG 2 failed to reach the genome-wide significance threshold. QTLpoly predicted a two-QTL model, detecting peaks on LG 3 and 4. A putative QTL was initially detected on LG 6 in the forward search (sig.fwd = 0.01), but was removed in the subsequent backward elimination step (sig.bwd = 0.0001). The major QTL on LG 3 and 4 have also been reported in previous studies in diploid rose populations (Crespel *et al.*, 2002; Linde *et al.*, 2006).

**Fig. 2.**
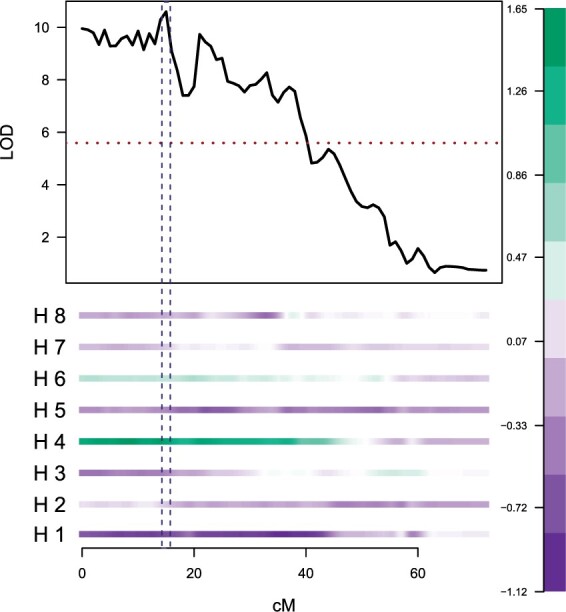
Exploration of the homologue effects at a QTL peak using polyqtlR. Example shown corresponds to the QTL peak position on LG 3 of tetraploid rose for the trait stem prickles. Positive effects (increasing the number of prickles) are coloured green, while negative effects are coloured purple. Parental homologues are numbered H1–H4 (maternal) and H5–H8 (paternal)

For the major QTL detected on LG 3, an additive model with QTL alleles for increased number of prickles located on parental homologues 4 and 6 (i.e. parental genotypes oooQ×oQoo) was found to have the lowest BIC of the 224 QTL models tested (listed in Supplementary Table S1), which corresponded well with the visualized homologue effects for that linkage group (Fig. 2).

We also used polyqtlR and QTLpoly to analyse an example trait ‘T32’ for a hexaploid population (TetraploidSNPMap is restricted to analyses of tetraploid populations and therefore was not included in this comparison). ‘T32’ is a simulated trait provided with the QTLpoly package for test purposes (Supplementary Data S1). Two peaks were detected in the initial genome-wide scan using polyqtlR, while a third peak became apparent after the major LG 1 peak was fitted as a co-factor (Fig. 1). The PVE of the three-QTL model was 50%. The location of the three peaks corresponded very closely to the true positions of the simulated QTL, with all three true QTL positions contained in the LOD—1 support intervals around the detected peaks. Indeed, polyqtlR detected all three simulated QTL with slightly higher precision than QTLpoly: the deviations between peak and true position for QTL 1a, 1b and 2 were 0.97, 7.98 and 0.99 cM for polyqtlR, while those for QTLpoly were 1.04, 9.26 and 1.19 cM, respectively.

### 3.2 Analysis of meiosis

**Fig. 3.**
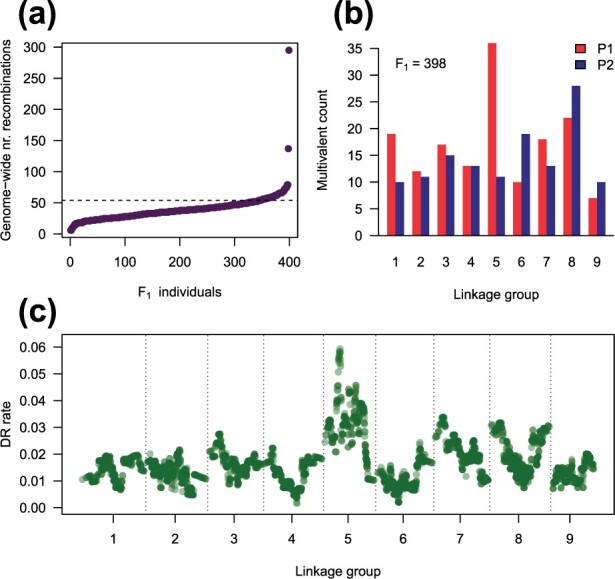
Explorations and visualizations of meiotic dynamics using polyqtlR. (**a**) Genome-wide counts of predicted recombinations per individual in a hexaploid chrysanthemum F_1_ population, derived from (bivalent-only) IBD probabilities with an error prior of 0.01. The horizontal dotted line shows the expected number of counts assuming on average one cross-over recombination per homologue (6×9 = 54); (**b**) Numbers of multivalent pairing structures predicted by the HMM per linkage group (*x*-axis). Maternal counts are shown in red (P1) while paternal counts are shown in blue (P2). Two F_1_ individuals with unusually high numbers of predicted recombinations in (a) were removed; (**c**) Rate of double reduction across the genome, using the same data as (b)

A hexaploid chrysanthemum (*Chrysanthemum*×*morifolium*) F_1_ population that had previously been used to generate a high-density linkage map (Van Geest *et al.*, 2017a) was re-analysed to gain insights into the parental meiosis. An analysis of the genome-wide counts of recombinations across the population showed that the dataset was of remarkably high quality (apart from a pair of outlying individuals), while the parental meioses appear to have involved fewer than the expected average of one cross-over event per bivalent (Fig. 3a).

The two outlier individuals were subsequently found to contain significantly more missing values that the rest of the population (Supplementary Fig. S1). They were removed and the remaining 398 individuals were re-analysed using the multivalent-aware HMM, allowing the number of multivalents per linkage group to be estimated (Fig. 3b). Maternal LG 5 had an unusually high number of predicted multivalents, which was reflected in a relatively high rate of predicted double reduction events for that chromosome, up to 6% (Fig. 3c).

**Fig. 4.**
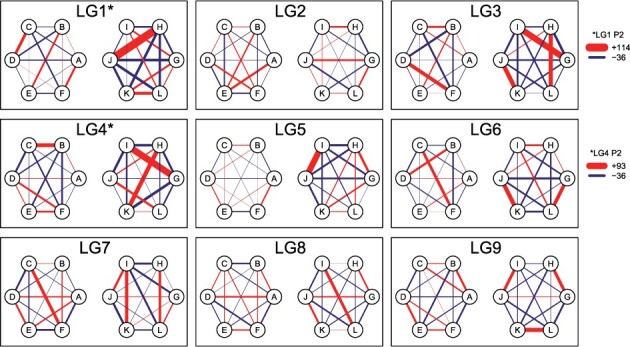
Deviations from a random-pairing model detected in a hexaploid chrysanthemum F_1_ population using polyqtlR. Maternal homologues are labelled A–F, while paternal homologues are labelled G–L (these labels are randomly assigned). The thickness of the line connecting parental homologues indicates the level of deviation from a random-pairing model, with counts exceeding expected proportions coloured red, and counts less than the expected proportions coloured blue

With multivalents accounted for, the remaining bivalent pairing configurations were used to test for preferential chromosome pairing. Deviations from a random-pairing (polysomic) model were tested using a chi-square test on the predicted counts of each set of bivalents per homologue (e.g. a test on the counts of AB, AC, AD, AE and AF for homologue A, etc.), while the deviations themselves can be used to visualize the chromosomal pairing patterns of both parents using polyqtlR (Fig. 4). There appeared to be evidence of non-random pairing in the paternal meiosis, with the most extreme deviation identified between paternal homologues H and J of linkage group 1. These homologues were predicted to have paired in 192 of the 389 bivalent-only meioses, an excess of 114 over the number expected if pairing were random (associated chi-square *P*-values of 2.8×10^−44^ and 5.4×10^−45^ for homologues H and J, respectively). For a number of chromosomes, three of the fifteen possible bivalent configurations were over-represented, for example in LG 3, 9 (and to a lesser extent LG 4 and 6) of parent 2 (Fig. 4). In all such cases (particularly for LG 3 and LG 9), the preferential bivalent pairings were complementary (i.e. involving all 6 homologues).

### 3.3 Speed and accuracy of IBD estimation

**Fig. 5.**
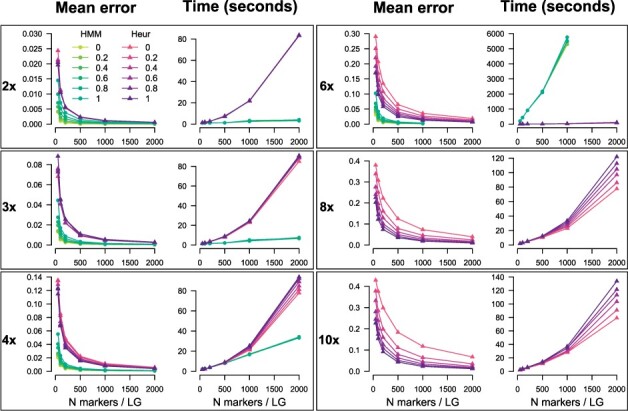
Mean error and computation time associated with estimation of IBD probabilities in polyqtlR. Comparison between results from the available options within the package: either a HMM or a heuristic algorithm (Heur). In each simulation, 5 chromosomes were simulated for a population of 200 individuals. Mean error was calculated as the average deviation in parental homologue probabilities from the true inheritance probabilities over all estimated positions and individuals. The legend (top left panel) refers to the proportion of simplex×nulliplex markers in the simulated datasets. For higher ploidy levels (8×, 10×), the HMM method has not been implemented and so no comparison was possible

The polyqtlR package contains two methods to estimate IBD probabilities which are used in many subsequent analyses. We critically compared these methods in terms of their accuracy and computational efficiency. F_1_ populations of 200 offspring each were simulated using PedigreeSim (Voorrips and Maliepaard, 2012) for ploidy levels 2×, 3×, 4×, 6×, 8× and 10×. A range of marker densities were simulated (50, 100, 200, 500, 1000 and 2000 markers per chromosome over 5 chromosomes), as well as differing proportions of simplex×nulliplex markers (proportions from 0 to 1 in steps of 0.2, where ‘0’ contained no 1× 0 or 0×1 markers, and ‘1’ contained 50% 1×0 and 50% 0×1 markers). As multivalents were not simulated, these were also not included in the IBD estimation. Computations were performed on a desktop PC (Intel Xeon processor, 3.6 GHz and 16 Gb RAM) in parallel over 5 cores. At lower ploidy levels (2×, 3× and 4×), the HMM was found to be both faster and more accurate than the heuristic method (Fig. 5), while at the hexaploid level the HMM was more accurate but had a high computational cost. Hexaploid datasets containing 10 000 markers (2000 per chromosome) were not analysed with the HMM due to protracted run-times. While datasets with higher proportions of simplex×nulliplex markers led to more accurate results using the heuristic method, the opposite was true of the HMM approach (Fig. 5). Regardless of the method used, both the error rate and computation time increased with increasing ploidy.

## 4 Discussion

We are currently witnessing an unprecedented number of developments in polyploid genomics, aided by increasingly affordable genotyping possibilities as well as the realization among breeders and researchers that genomics-assisted breeding in polyploid species is no longer an insurmountable challenge (Bourke *et al.*, 2018c; Smulders *et al.*, 2019). The polyqtlR package aims to facilitate this process by offering a range of tools to help uncover both the origin of favourable (or unfavourable) parental alleles for traits of interest, while also shedding light on how these alleles are passed from one generation to the next by exploring meiotic dynamics of polyploid species.

### 4.1 Software alternatives and QTL validation

It is usual for new software tools to compare their performance to previously released software alternatives. We have nominally done so by comparing the results of polyqtlR to existing packages TetraploidSNPMap and QTLpoly for traits in tetraploid and hexaploid populations, but without quantifying performance differences. The use of the additive-effect interval mapping approach in polyqtlR and TetraploidSNPMap is less computationally expensive than fitting mixed models as is done in QTLpoly, and this was indeed reflected in the run-times we observed. For the trait stem prickles in the tetraploid rose population, polyqtlR detected four putative QTL, three of which were confirmed by TetraploidSNPMap and two of which were confirmed by QTLpoly and were also detected in independent experiments with diploid rose populations (Crespel *et al.*, 2002; Linde *et al.*, 2006). We feel this demonstrates the importance of comparing results of various software. The peak on LG 2 that we detected may possibly have been a ‘false positive’ detection, although in exploratory analyses these may be less of a concern than possible ‘false negatives’, such as the LG 6 peak that was eliminated in subsequent mapping rounds by QTLpoly. It is interesting to note that the precision of polyqtlR for the simulated trait ‘T32’ in the hexaploid population was slightly higher than QTLpoly (all three QTL peak positions were closer to the true QTL positions). This trait has previously been used to demonstrate the superiority of multi-QTL models over single-QTL ones (Pereira *et al.*, 2019), while we have demonstrated here that a ‘fixed effect interval mapping’ approach is equally capable of building an accurate multi-QTL model if genetic co-factors are included.

Finally, an earlier version of polyqtlR was previously tested in an investigation of the effects of double reduction on QTL detection (Bourke *et al.*, 2019). QTL for the traits flesh colour and plant maturity in tetraploid potato coincided with known underlying genes *StCDF1* (Kloosterman *et al.*, 2013) and *StChy2* (Wolters *et al.*, 2010), while a large simulation study confirmed the ability of the package to accurately identify simulated QTL under a wide range of parameter settings (Bourke *et al.*, 2019).

### 4.2 Hexaploid inheritance

Using polyqtlR, we uncovered evidence of preferential pairing in hexaploid chrysanthemum, a phenomenon that was not detected in a previous study using the same population and genotypes (Van Geest *et al.*, 2017b). This highlights the power of leveraging map and genotype information to correctly diagnose preferential pairing, as was done previously in a study of tetraploid rose (Bourke *et al.*, 2017). From our analysis it appears that the hexaploid parents of this population indeed exhibited predominantly hexasomic inheritance, but with some clear exceptions to this trend (Fig. 4). Attempting to parametrize preferential pairing at the hexaploid level or higher is clearly non-trivial given the variable patterns of preferential pairing observed here. In some cases, a single pair of homologues behaved as a ‘sub-genomic unit’ (i.e. showing a strong pairing preference), while elsewhere in the genome, multiple sets of complementary homologue pairs showed non-random pairing patterns, reminiscent of a more allopolyploid-like pairing behaviour for these chromosomes (Fig. 4). These sorts of insights could potentially be of enormous importance to breeders aiming to recombine specific alleles on a single homologue (in coupling phase). These meiotic insights are also of fundamental interest to biologists, providing experimental evidence regarding the mode of inheritance of polyploid species that may not fall neatly into the categories of allo- or autopolyploid (Bourke *et al.*, 2017).

### 4.3 Innovative aspects

polyqtlR offers a number of innovations not available elsewhere. For example, it allows the inclusion of multivalent structures in the inheritance model for triploid, tetraploid and hexaploid populations, carried through to subsequent QTL analyses and explorations of parental meioses. At the hexaploid level this is unique, allowing us to estimate rates of multivalent pairing and visualize the double reduction landscape, something that to the best of our knowledge has never previously been visualized in a hexaploid species (Fig. 3). The practical implications of double reduction events for QTL mapping may be relatively minor (Bourke *et al.*, 2019), but they can have potentially important breeding implications by increasing the frequency of favourable alleles in particular individuals, as well as being of theoretical interest to polyploid geneticists.

The package also calculates and visualizes per-homologue profiles of the GIC, one of the major factors determining QTL detection power and precision (Bourke *et al.*, 2019). Through visual inspection, parental homologues with poor information can be easily identified and potentially targeted with additional markers.

Options for genotype curation are relatively limited for polyploid species currently, but can be achieved in polyqtlR through IBD-informed genotype imputation. The choice of a suitable error prior ε in IBD estimation is critical to this step. ε is not known *a priori*, but can be estimated *a posteriori* by running the IBD estimation step a number of times with different values (e.g. ε = 0.01, 0.05, 0.1 and 0.2) and comparing the marginal likelihoods across the mapping population between runs. Applying a higher error prior (e.g. ε=0.2) makes transitions between states less probable in the HMM procedure, thus penalizing multiple cross-overs that are often necessary to accommodate genotyping errors in a predicted meiotic model with an overly conservative error prior. These can be used directly to re-impute marker genotypes, which could subsequently be used in re-estimating linkage maps that may have been built under the assumption of error-free data.

polyqtlR also includes a heuristic approach to IBD probability estimation, something that is not currently available elsewhere but which allows IBD probabilities to be approximated in a relatively short time for populations of *all* ploidy levels, with almost no increase in computation time with increasing ploidy level (Fig. 5). Our approach to detecting and visualizing preferential chromosome pairing (Fig. 4) also provides a clear overview of meiotic pairing and recombination dynamics across experimental populations, leading to insights into pairing behaviour at a level of detail not previously possible. Finally, although not demonstrated here, polyqtlR can identify recombinant individuals for specific homologues, a functionality that could be used for tailored breeding approaches or ‘breeding-by-design’ for polyploid crops (Peleman and Van Der Voort, 2003).

### 4.4 Concluding remarks

In this paper we have introduced a novel R package to facilitate QTL analysis and the exploration of chromosomal pairing in polyploid species. polyqtlR is freely available under the general public license from the Comprehensive R Archive Network (CRAN) at http://cran.r-project.org/package=polyqtlR.

## Supplementary Material

btab574_Supplementary_Data

## References

[btab574-B1] Blakeslee A.F. , Avery A.G. (1937) Methods of inducing doubling of chromosomes in plants: by treatment with colchicine. J. Hered., 28, 393–411.

[btab574-B2] Bourke P.M. (2014) QTL analysis in polyploids. MSc Thesis, Wageningen University, Wageningen.

[btab574-B3] Bourke P.M. et al (2017) Partial preferential chromosome pairing is genotype dependent in tetraploid rose. Plant J., 90, 330–343. 28142191 10.1111/tpj.13496

[btab574-B4] Bourke P.M. et al (2015) The double reduction landscape in tetraploid potato as revealed by a high-density linkage map. Genetics, 201, 853–863. 26377683 10.1534/genetics.115.181008 PMC4649655

[btab574-B5] Bourke P.M. et al (2018a) Multi-environment QTL analysis of plant and flower morphological traits in tetraploid rose. Theor. Appl. Genet., 131, 2055–2069. 29961102 10.1007/s00122-018-3132-4 PMC6154034

[btab574-B6] Bourke P.M. et al (2018b) polymapR: linkage analysis and genetic map construction from F1 populations of outcrossing polyploids. Bioinformatics, 34, 3496–3502. 29722786 10.1093/bioinformatics/bty371 PMC6184683

[btab574-B7] Bourke P.M. et al (2018c) Tools for genetic studies in experimental populations of polyploids. Front. Plant Sci., 9, 513. 29720992 10.3389/fpls.2018.00513 PMC5915555

[btab574-B8] Bourke P.M. et al (2019) Quantifying the power and precision of QTL analysis in autopolyploids under bivalent and multivalent genetic models. G3 Genes Genomes Genet., 9, 2107–2122. 10.1534/g3.119.400269 PMC6643892 31036677

[btab574-B9] Bradshaw J.E. (2016) Plant Breeding: Past, Present and Future. Springer, New York.

[btab574-B10] Broman K.W. , Sen S. (2009) A Guide to QTL Mapping with R/qtl. Springer, New York.

[btab574-B11] Carley CaS. et al (2017) Automated tetraploid genotype calling by hierarchical clustering. Theor. Appl. Genet., 130, 717–726. 28070610 10.1007/s00122-016-2845-5

[btab574-B12] Churchill G.A. , Doerge R.W. (1994) Empirical threshold values for quantitative trait mapping. Genetics, 138, 963–971. 7851788 10.1093/genetics/138.3.963 PMC1206241

[btab574-B13] Clark L.V. et al (2019) polyRAD: genotype calling with uncertainty from sequencing data in polyploids and diploids. G3 Genes Genomes Genet., 9, 663–673. 10.1534/g3.118.200913 PMC6404598 30655271

[btab574-B14] Comai L. (2005) The advantages and disadvantages of being polyploid. Nat. Rev. Genet., 6, 836–846. 16304599 10.1038/nrg1711

[btab574-B15] Crespel L. et al (2002) Mapping of qualitative and quantitative phenotypic traits in *Rosa* using AFLP markers. Theor. Appl. Genet., 105, 1207–1214. 12582900 10.1007/s00122-002-1102-2

[btab574-B16] Gerard D. et al (2018) Genotyping polyploids from messy sequencing data. Genetics, 210, 789–807. 30185430 10.1534/genetics.118.301468 PMC6218231

[btab574-B17] Hackett C.A. et al (2017) TetraploidSNPMap: software for linkage analysis and QTL mapping in autotetraploid populations using SNP dosage data. J. Hered., 108, 438–442.

[btab574-B18] Hackett C.A. et al (2014) QTL mapping in autotetraploids using SNP dosage information. Theor. Appl. Genet., 127, 1885–1904. 24981609 10.1007/s00122-014-2347-2 PMC4145212

[btab574-B19] Hackett C.A. et al (2013) Linkage analysis and QTL mapping using SNP dosage data in a tetraploid potato mapping population. PLoS One, 8, e63939. 23704960 10.1371/journal.pone.0063939 PMC3660524

[btab574-B20] Herben T. et al (2017) Polyploid species rely on vegetative reproduction more than diploids: a re-examination of the old hypothesis. Ann. Bot., 120, 341–349. 28334206 10.1093/aob/mcx009 PMC5737615

[btab574-B21] Jansen R. (1992) A general mixture model for mapping quantitative trait loci by using molecular markers. Theor. Appl. Genet., 85, 252–260. 24197312 10.1007/BF00222867

[btab574-B22] Jansen R.C. (1993) Interval mapping of multiple quantitative trait loci. Genetics, 135, 205–211. 8224820 10.1093/genetics/135.1.205 PMC1205619

[btab574-B23] Kempthorne O. (1957). An Introduction to Genetic Statistics. John Wiley & Sons, New York.

[btab574-B24] Kloosterman B. et al (2013) Naturally occurring allele diversity allows potato cultivation in northern latitudes. Nature, 495, 246–250. 23467094 10.1038/nature11912

[btab574-B25] Leal‐Bertioli S.C. et al (2018) Segmental allopolyploidy in action: increasing diversity through polyploid hybridization and homoeologous recombination. Am. J. Bot., 105, 1053–1066. 29985538 10.1002/ajb2.1112

[btab574-B26] Levin D.A. (1983) Polyploidy and novelty in flowering plants. Am. Nat., 122, 1–25.

[btab574-B27] Linde M. et al (2006) Powdery mildew resistance in roses: QTL mapping in different environments using selective genotyping. Theor. Appl. Genet., 113, 1081–1092. 16896710 10.1007/s00122-006-0367-2

[btab574-B28] Mollinari M. , Garcia AF. (2019) Linkage analysis and haplotype phasing in experimental autopolyploid populations with high ploidy level using hidden Markov models. G3 Genes Genomes Genetics, 9, 3297–3314. 31405891 10.1534/g3.119.400378 PMC6778803

[btab574-B29] Peleman J.D. , Van Der Voort J.R. (2003) Breeding by design. Trends Plant Sci., 8, 330–334. 12878017 10.1016/S1360-1385(03)00134-1

[btab574-B30] Pereira G.D. et al (2020) Multiple QTL mapping in autopolyploids: a random-effect model approach with application in a hexaploid sweetpotato full-sib population. Genetics, 215, 579–595. 32371382 10.1534/genetics.120.303080 PMC7337090

[btab574-B31] Pereira G.D. et al (2019) *Tutorial on Multiple QTL Mapping in Autopolyploids with QTLpoly*. https://guilherme-pereira.github.io/QTLpoly/1-tutorial (1 February 2021, date last accessed).

[btab574-B32] Pereira G.S. et al (2018) A fully automated pipeline for quantitative genotype calling from next generation sequencing data in autopolyploids. BMC Bioinformatics, 19, 1–10. 30382832 10.1186/s12859-018-2433-6 PMC6211426

[btab574-B33] Pinheiro J. et al; R Core Team. (2017) *nlme: Linear and Nonlinear Mixed Effects Models. R package version 3.1-131*. https://cran.r-project.org/web/packages/nlme/citation.html.

[btab574-B34] R Core Team (2020). R: A Language and Environment for Statistical Computing. R Foundation for Statistical Computing, Vienna, Austria.

[btab574-B35] Renny-Byfield S. , Wendel J.F. (2014) Doubling down on genomes: polyploidy and crop plants. Am. J. Bot., 101, 1711–1725. 25090999 10.3732/ajb.1400119

[btab574-B36] Salman-Minkov A. et al (2016) Whole-genome duplication as a key factor in crop domestication. Nat. Plants, 2, 16115. 27479829 10.1038/nplants.2016.115

[btab574-B37] Sattler M.C. et al (2016) The polyploidy and its key role in plant breeding. Planta, 243, 281–296. 26715561 10.1007/s00425-015-2450-x

[btab574-B38] Schwarz G. (1978) Estimating the dimension of a model. Ann. Stat., 6, 461–464.

[btab574-B39] Serang O. et al (2012) Efficient exact maximum a posteriori computation for Bayesian SNP genotyping in polyploids. PLoS One, 7, e30906. 22363513 10.1371/journal.pone.0030906 PMC3281906

[btab574-B40] Smulders M.J. et al (2019) In the name of the rose: a roadmap for rose research in the genome era. Horticulture Res., 6, 1–17. 10.1038/s41438-019-0156-0 PMC6499834 31069087

[btab574-B41] te Beest M. et al (2012) The more the better? The role of polyploidy in facilitating plant invasions. Ann. Bot., 109, 19–45. 22040744 10.1093/aob/mcr277 PMC3241594

[btab574-B42] Udall J.A. , Wendel J.F. (2006) Polyploidy and crop improvement. Crop Sci., 46, S–3.

[btab574-B43] Van Geest G. et al (2017a) An ultra-dense integrated linkage map for hexaploid chrysanthemum enables multi-allelic QTL analysis. Theor. Appl. Genet., 130, 2527–2541. 28852802 10.1007/s00122-017-2974-5 PMC5668331

[btab574-B44] Van Geest G. et al (2017b) Conclusive evidence for hexasomic inheritance in chrysanthemum based on analysis of a 183 k SNP array. BMC Genomics, 18, 1–12. 28784083 10.1186/s12864-017-4003-0 PMC5547472

[btab574-B45] Van Ooijen J. (2009) MapQTL ^®^ 6, Software for the Mapping of Quantitative Trait Loci in Experimental Populations of Diploid Species. Kyazma B.V., Wageningen, Netherlands.

[btab574-B46] Van Ooijen J.W. (1992) Accuracy of mapping quantitative trait loci in autogamous species. Theor. Appl. Genet., 84, 803–811. 24201478 10.1007/BF00227388

[btab574-B47] Van Tuyl J.M. , Lim K.B. (2003) Interspecific hybridisation and polyploidisation as tools in ornamental plant breeding. Acta Horticulturae, 612, 13–22.

[btab574-B48] Vanneste K. et al (2014) Analysis of 41 plant genomes supports a wave of successful genome duplications in association with the Cretaceous–Paleogene boundary. Genome Res., 24, 1334–1347. 24835588 10.1101/gr.168997.113 PMC4120086

[btab574-B49] Voorrips R.E. et al (2011) Genotype calling in tetraploid species from bi-allelic marker data using mixture models. BMC Bioinformatics, 12, 172. 21595880 10.1186/1471-2105-12-172 PMC3121645

[btab574-B50] Voorrips R.E. , Maliepaard C.A. (2012) The simulation of meiosis in diploid and tetraploid organisms using various genetic models. BMC Bioinformatics, 13, 248. 23013469 10.1186/1471-2105-13-248 PMC3542247

[btab574-B51] Wolters A.-M.A. et al (2010) Identification of alleles of carotenoid pathway genes important for zeaxanthin accumulation in potato tubers. Plant Mol. Biol., 73, 659–671. 20490894 10.1007/s11103-010-9647-y PMC2898108

[btab574-B52] Zheng C. et al (2020) Haplotype reconstruction in connected tetraploid F1 populations. bioRxiv, doi: 10.1101/2020.12.18.423519. 10.1093/genetics/iyab106 PMC8633103 34849879

[btab574-B53] Zheng C. et al (2016) Probabilistic multilocus haplotype reconstruction in outcrossing tetraploids. Genetics, 203, 119–131. 26920758 10.1534/genetics.115.185579 PMC4858767

[btab574-B54] Zych K. et al (2019) FitTetra 2.0–improved genotype calling for tetraploids with multiple population and parental data support. BMC Bioinformatics, 20, 1–8. 30894135 10.1186/s12859-019-2703-y PMC6425654

