# Supplementary material for: Detecting quantitative trait loci and exploring chromosomal pairing in autopolyploids using polyqtlR
Source: Bioinformatics. 2021 Aug 6;37(21):3822–9. doi: 10.1093/bioinformatics/btab574 (PMC8570814; doi:10.1093/bioinformatics/btab574)
Supplement: btab574_Supplementary_Data [file btab574_supplementary_data.zip › Supplementary Methods.docx]

## Supplementary Methods

### SM1. Estimation of IBD probabilities using HMM

We largely followed the approach (and notation) described by Zheng et al. (2016). For diploids, only bivalent pairing needs to be considered, with the 2 x 2 transition probability matrix $\tau_{{[c}_{1}c_{2}]}$ between homologous chromosomes $c_{1}$ and $c_{2}$ given by

$$\tau_{{[c}_{1}c_{2}]}=\left[ \begin{matrix} 1-r & r \\ r & 1-r \end{matrix} \right]$$

where *r* is the recombination frequency between adjacent markers derived from a genetic map. For diploids, $\tau_{{[c}_{1}c_{2}]}$ also corresponds to the gamete transition matrix $T_{[c_{1}c_{2}]}$. For triploids, tetraploids and hexaploids, the gamete model can be specified either under the assumption of bivalent-only pairing or considering multivalent pairing (which may be applicable in the tetraploid parent for triploid populations).

#### Bivalent model

In the case of bivalent-only pairing, the gamete transition matrix for tetraploids is the Kronecker product of two bivalent transition probability matrices, *e.g.* ${T_{\left[ c_{1}c_{2} \right][c_{3}c_{4}]}= \tau}_{{[c}_{1}c_{2}]}\otimes\tau_{[c_{3}c_{4}]}$ for the case of homologue pairing [12][34], while in hexaploids the Kronecker product of three such transition probability matrices is required (*e.g.* ${T_{\left[ c_{1}c_{2} \right]\left[ c_{3}c_{4} \right][c_{5}c_{6}]}= \tau}_{{[c}_{1}c_{2}]}\otimes\tau_{[c_{3}c_{4}]}\otimes\tau_{[c_{5}c_{6}]}$). In a tetraploid there are three possible homologue pairing combinations, *i.e.* [12][34], [13][24] or [14][23] (Figure 1.a), while for a hexaploid the number of bivalent pairing combinations per parent increases to 15.

**Figure 1. Pairing structures in an autotetraploid.** **a.** The three possible homologue bivalent pairing combinations in a parental meiosis are shown: [12][34], [13][24] and [14][23].

**b.** Example of a multivalent (here, a quadrivalent) pairing structure. For visual clarity in both figures, pairs of sister chromatids are represented as single chromosomes rather than as bound pairs


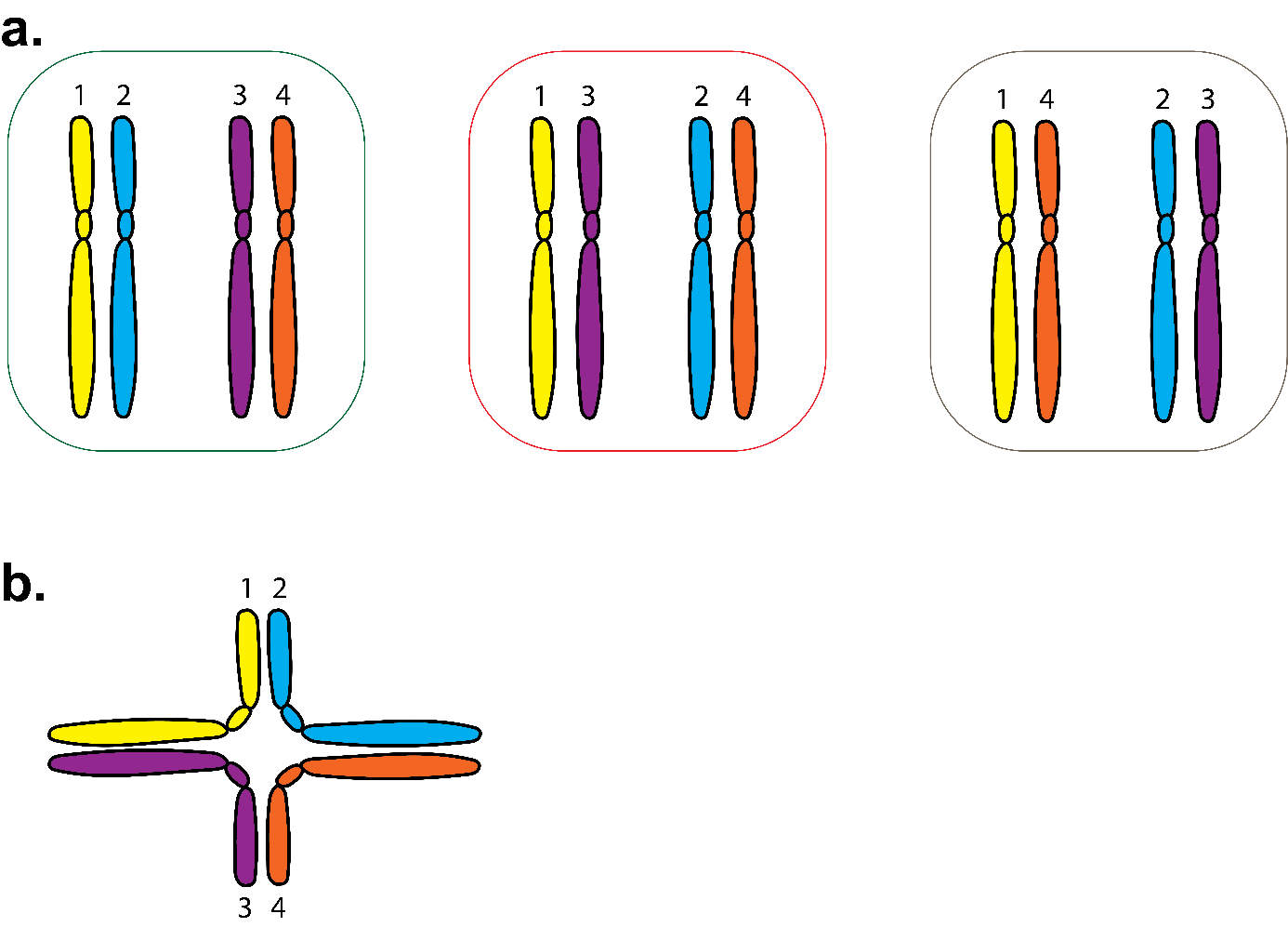


#### Multivalent model

A multivalent is a pairing structure involving more than two homologues (Figure 1.b), although structures containing an odd number of homologues are not considered as they generally do not lead to balanced gametes. The transition matrix for a multivalent is a generalisation of the bivalent case, as proposed by Zheng *et al.* (2016).

For tetraploids, it is given by $\tau_{\left[ 1234 \right]}=\left[ \begin{matrix} 1-r & \frac{r}{3} & \frac{r}{3} & \frac{r}{3} \\ \frac{r}{3} & 1-r & \frac{r}{3} & \frac{r}{3} \\ \frac{r}{3} & \frac{r}{3} & 1-r & \frac{r}{3} \\ \frac{r}{3} & \frac{r}{3} & \frac{r}{3} & 1-r \end{matrix} \right]$

while for hexaploids it is given by $\tau_{\left[ 123456 \right]}=\left[ \begin{matrix} 1-r & \frac{r}{5} & \frac{r}{5} & \frac{r}{5} & \frac{r}{5} & \frac{r}{5} \\ \frac{r}{5} & 1-r & \frac{r}{5} & \frac{r}{5} & \frac{r}{5} & \frac{r}{5} \\ \frac{r}{5} & \frac{r}{5} & 1-r & \frac{r}{5} & \frac{r}{5} & \frac{r}{5} \\ \frac{r}{5} & \frac{r}{5} & \frac{r}{5} & 1-r & \frac{r}{5} & \frac{r}{5} \\ \frac{r}{5} & \frac{r}{5} & \frac{r}{5} & \frac{r}{5} & 1-r & \frac{r}{5} \\ \frac{r}{5} & \frac{r}{5} & \frac{r}{5} & \frac{r}{5} & \frac{r}{5} & 1-r \end{matrix} \right]$

Note that in the case of hexaploids, the exact nature of the multivalent structure is not specified, although the transition matrix can account for both a quadrivalent + bivalent as well as a hexavalent pairing structure. This specification in theory allows an offspring to inherit a haplotype composed of segments of all 6 parental homologues (similarly in the tetraploid case a mosaic of 4 homologues is allowed by this formulation). The fact that this does not reflect biological reality need not concern us; the formulation is general enough to capture all the known features of polysomic inheritance, while the probability of incorrectly predicting such imaginary homologue mosaics is essentially zero. The gamete transition matrices accounting for multivalents for tetraploids and hexaploids are given by

$T_{[1234]}=\tau_{\left[ 1234 \right]}\otimes\tau_{\left[ 1234 \right]}$

and

$$T_{[123456]}=\tau_{\left[ 123456 \right]}\otimes\tau_{\left[ 123456 \right]}\otimes\tau_{\left[ 123456 \right]}$$

respectively.

The transition matrix of a particular offspring *o* (termed the “zygote model” (Zheng et al., 2016)) is given by the Kronecker product of both parental gamete transition matrices, for example $T^{o}=T_{\left[ 123456 \right]}\otimes T_{\left[ c_{7}c_{8} \right]\left[ c_{9}c_{10} \right][c_{11}c_{12}]}$ for the case of multivalent pairing in parent 1 and bivalent pairing in parent 2.

For a given ploidy and chromosomal pairing model, a set of chromosomal “valency” configurations are generated. In the case of tetraploids and hexaploids, this may be bivalents in both parents, a bivalent in one parent (and a multivalent in the other), or bivalents in neither parent (i.e. multivalent pairing in both parents). For hexaploids, the option of multivalent pairing in both parents at the same locus has been disabled by default, but can be enabled by setting the argument “full_multivalent_hexa” to TRUE, if sufficient memory is available. For the simplest case (bivalents in both parents), there are 9 such valencies to consider in a tetraploid (as there are three pairing combinations possible in each parent (Figure 1.a), leading to 3 x 3 = 9 combinations across both parents). Each valency is described by a 16 x 4 state matrix representing the 16 possible genotype combinations associated with that particular chromosomal pairing at a particular locus (Table 1). For example, with valency [12][34] | [56][78], genotype 1-3-5-7 (the combination of parental alleles 1, 3, 5 and 7) is contained in the state matrix, while 1-2-5-6 is not (Table 1, column 1).

**Table 1.** Example of the state matrices associated with each of the nine valencies (one per column) associated with bivalent-only pairing in a tetraploid x tetraploid cross

| **[12][34] \| [56][78]** | **[12][34] \| [57][68]** | **[12][34] \| [58][67]** | **[13][24] \| [56][78]** | **[13][24] \| [57][68]** | **[13][24] \| [58][67]** | **[14][23] \| [56][78]** | **[14][23] \| [57][68]** | **[14][23] \| [58][67]** |
| --- | --- | --- | --- | --- | --- | --- | --- | --- |
| 1-3-5-7 | 1-3-5-6 | 1-3-5-6 | 1-2-5-7 | 1-2-5-6 | 1-2-5-6 | 1-2-5-7 | 1-2-5-6 | 1-2-5-6 |
| 1-3-5-8 | 1-3-5-8 | 1-3-5-7 | 1-2-5-8 | 1-2-5-8 | 1-2-5-7 | 1-2-5-8 | 1-2-5-8 | 1-2-5-7 |
| 1-3-6-7 | 1-3-6-7 | 1-3-6-8 | 1-2-6-7 | 1-2-6-7 | 1-2-6-8 | 1-2-6-7 | 1-2-6-7 | 1-2-6-8 |
| 1-3-6-8 | 1-3-7-8 | 1-3-7-8 | 1-2-6-8 | 1-2-7-8 | 1-2-7-8 | 1-2-6-8 | 1-2-7-8 | 1-2-7-8 |
| 1-4-5-7 | 1-4-5-6 | 1-4-5-6 | 1-4-5-7 | 1-4-5-6 | 1-4-5-6 | 1-3-5-7 | 1-3-5-6 | 1-3-5-6 |
| 1-4-5-8 | 1-4-5-8 | 1-4-5-7 | 1-4-5-8 | 1-4-5-8 | 1-4-5-7 | 1-3-5-8 | 1-3-5-8 | 1-3-5-7 |
| 1-4-6-7 | 1-4-6-7 | 1-4-6-8 | 1-4-6-7 | 1-4-6-7 | 1-4-6-8 | 1-3-6-7 | 1-3-6-7 | 1-3-6-8 |
| 1-4-6-8 | 1-4-7-8 | 1-4-7-8 | 1-4-6-8 | 1-4-7-8 | 1-4-7-8 | 1-3-6-8 | 1-3-7-8 | 1-3-7-8 |
| 2-3-5-7 | 2-3-5-6 | 2-3-5-6 | 2-3-5-7 | 2-3-5-6 | 2-3-5-6 | 2-4-5-7 | 2-4-5-6 | 2-4-5-6 |
| 2-3-5-8 | 2-3-5-8 | 2-3-5-7 | 2-3-5-8 | 2-3-5-8 | 2-3-5-7 | 2-4-5-8 | 2-4-5-8 | 2-4-5-7 |
| 2-3-6-7 | 2-3-6-7 | 2-3-6-8 | 2-3-6-7 | 2-3-6-7 | 2-3-6-8 | 2-4-6-7 | 2-4-6-7 | 2-4-6-8 |
| 2-3-6-8 | 2-3-7-8 | 2-3-7-8 | 2-3-6-8 | 2-3-7-8 | 2-3-7-8 | 2-4-6-8 | 2-4-7-8 | 2-4-7-8 |
| 2-4-5-7 | 2-4-5-6 | 2-4-5-6 | 3-4-5-7 | 3-4-5-6 | 3-4-5-6 | 3-4-5-7 | 3-4-5-6 | 3-4-5-6 |
| 2-4-5-8 | 2-4-5-8 | 2-4-5-7 | 3-4-5-8 | 3-4-5-8 | 3-4-5-7 | 3-4-5-8 | 3-4-5-8 | 3-4-5-7 |
| 2-4-6-7 | 2-4-6-7 | 2-4-6-8 | 3-4-6-7 | 3-4-6-7 | 3-4-6-8 | 3-4-6-7 | 3-4-6-7 | 3-4-6-8 |
| 2-4-6-8 | 2-4-7-8 | 2-4-7-8 | 3-4-6-8 | 3-4-7-8 | 3-4-7-8 | 3-4-6-8 | 3-4-7-8 | 3-4-7-8 |

The likelihood of each particular valency is estimated using the Forward-Backward algorithm (Durbin et al., 1998). Assuming a known parental marker phasing (*e.g.* as the outcome of a linkage map), emission matrices for the Markov chain are generated by comparing per offspring its dosage score at a marker position with the summed parental scores from the phased map, summed across terms given by the state matrix for that valency. For example in a tetraploid, if the known parental phasing at a marker was 1001 | 0011 and the assumed (but as yet unknown) offspring genotype was 1-2-5-8 (a row in the state matrix), then an offspring dosage of 1+0+0+1 = 2 would be compatible, while all other observed dosage scores (0, 1, 3, 4) would be considered incompatible. A genome-wide error prior $\varepsilon$ is used to account for genotyping errors, with compatible offspring scores assigned a probability of $1-\varepsilon$ and all other dosages classes assigned a probability of $\frac{\varepsilon}{ploidy}$ . In the case of a missing genotyping score, a probability of $\frac{1}{N_{states}}$ is assigned, where $N_{states}$ is the number of rows of the state matrix being considered. For probabilistic genotypes, determining a compatible or incompatible offspring score is less clear than for discrete genotypes. We chose to consider an observation compatible if the offspring genotype probability for that score exceeded 0.5. Posterior offspring probabilities are calculated using the product of the forward and backward probability matrices, and the log probability from the termination step is recorded to identify the valency with the highest likelihood. The final steps in generating offspring genotype (IBD) probabilities are as described in Zheng *et al.* (2016).

### SM2. Estimation of IBD probabilities using Heuristic algorithm

A simple approach to approximate IBD probabilities without resort to hidden Markov models has also been implemented in the package to facilitate high-speed analyses, which is of particular relevance at higher ploidy levels. IBD probabilities of 0.5 are initially assigned to the population for all parental homologues at all marker positions (completely uninformative priors). Fully-informative marker scores are then used to assign probabilities of 1 in the case of inheritance, or 0 in the case of no inheritance (Figure 2).


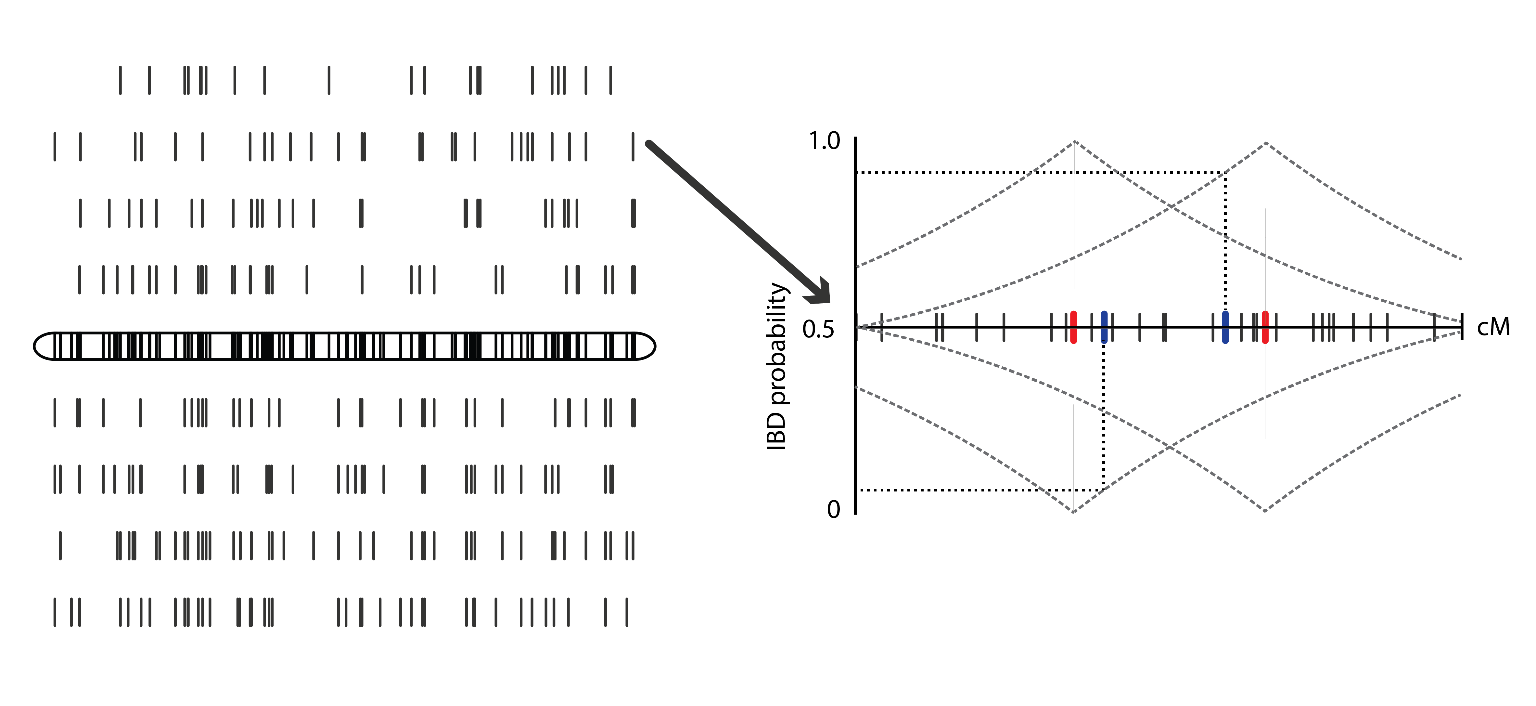


**Figure 2. Heuristic approach to IBD probability estimation in *polyqtlR*.** A phased linkage map is shown on the left, with four homologues of each tetraploid parent and the integrated chromosomal map in the centre. For each parental homologue, a set of informative markers are identified (shown in red here, in simplex condition in that parent) and are used to assign IBD probabilities per individual first at the locus itself, and subsequently to flanking loci which are non-informative in that parent (two example markers are shown in blue). Marker presence in an individual results in an assigned probability of 1 (and associated upper curves), while marker absence results in an assigned probability of 0 (and associated lower curves). The rate of decay of IBD haplotype probability is modelled using the appropriate inverse mapping function (*e.g.* Haldane’s) as used in map construction.

For example, in a tetraploid population at a 1x0 marker (single segregating allele originating from parent 1) which was phased 0001 | 0000, offspring with marker dosage 1 would initially be assigned probabilities of (0.5,0.5,0.5,**1** | 0.5,0.5,0.5,0.5), and offspring with marker dosage 0 would initially be assigned probabilities of (0.5,0.5,0.5,**0** | 0.5,0.5,0.5,0.5) (normalisation of probabilities occurs later).

­­Partially-informative dosages in the offspring are not used (*e.g.* an offspring dosage of 1 from a 1x1 marker – the origin of this allele could be from either parent). Once all such probabilities have been assigned, probabilities at all other marker positions are approximated by first locating the nearest flanking marker with an informative probability (0 or 1). The probability is then estimated as *r* in the case of a starting probability of 0, or 1 – *r* in the case of a starting probability of 1, where *r* is the recombination frequency between the two markers, as derived from the map positions (for example, if Haldane’s mapping function (Haldane, 1919) was used to generate the map, the inverse of this function should be used to re-calculate *r*). The IBD probabilities $P_{i}$ are then normalised to ensure $\sum_{i} P_{i}= \frac{ploidy}{2}$ for each parent.

## References

Durbin, R., Eddy, S.R., Krogh, A., and Mitchison, G. (1998). *Biological sequence analysis: probabilistic models of proteins and nucleic acids.* Cambridge university press.

Haldane, J. (1919). The combination of linkage values and the calculation of distances between the loci of linked factors. *J Genet* **8,** 299-309.

Zheng, C., Voorrips, R.E., Jansen, J., Hackett, C.A., Ho, J., and Bink, M.C. (2016). Probabilistic Multilocus Haplotype Reconstruction in Outcrossing Tetraploids. *Genetics* **203,** 119-131.
