# Supplementary figures and images for: Detecting quantitative trait loci and exploring chromosomal pairing in autopolyploids using polyqtlR
Source: Bioinformatics. 2021 Aug 6;37(21):3822–9. doi: 10.1093/bioinformatics/btab574 (PMC8570814; doi:10.1093/bioinformatics/btab574)

Nr. predicted recombinations

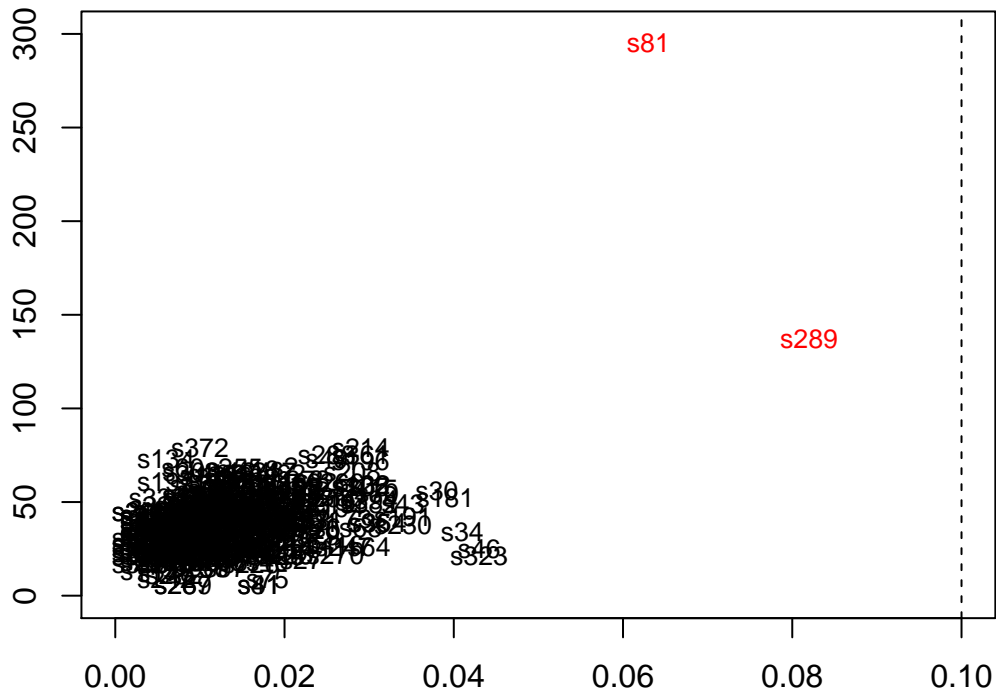

NA rate

Supplement: btab574_Supplementary_Data [file btab574_supplementary_data.zip › Figure_S1.pdf]
